# Host-Directed Therapy as a Novel Treatment Strategy to Overcome Tuberculosis: Targeting Immune Modulation

**DOI:** 10.3390/antibiotics9010021

**Published:** 2020-01-07

**Authors:** Sultan Ahmed, Rubhana Raqib, Guðmundur Hrafn Guðmundsson, Peter Bergman, Birgitta Agerberth, Rokeya Sultana Rekha

**Affiliations:** 1 Department of Laboratory Medicine, Division of Clinical Microbiology, Karolinska Institutet, 141 52 Stockholm, Sweden; sultan.ahmed@ki.se (S.A.); ghrafn@hi.is (G.H.G.); Peter.Bergman@ki.se (P.B.); Birgitta.Agerberth@ki.se (B.A.); 2 Infectious Diseases Division, International Centre for Diarrhoeal Disease Research, Bangladesh (icddr,b), Dhaka 1213, Bangladesh; rubhana@icddrb.org; 3 Biomedical Center, University of Iceland, 101 Reykjavik, Iceland; 4 Infectious Disease Clinic, the Immunodeficiency Unit, Karolinska University Hospital, 141 52 Stockholm, Sweden

**Keywords:** *Mycobacterium tuberculosis*, host-directed therapy, drug resistance, immune response, autophagy, innate immunity, antimicrobial peptides

## Abstract

Tuberculosis (TB) is one of the leading causes of mortality and morbidity, particularly in developing countries, presenting a major threat to the public health. The currently recommended long term treatment regimen with multiple antibiotics is associated with poor patient compliance, which in turn, may contribute to the emergence of multi-drug resistant TB (MDR-TB). The low global treatment efficacy of MDR-TB has highlighted the necessity to develop novel treatment options. Host-directed therapy (HDT) together with current standard anti-TB treatments, has gained considerable interest, as HDT targets novel host immune mechanisms. These immune mechanisms would otherwise bypass the antibiotic bactericidal targets to kill *Mycobacterium tuberculosis* (Mtb), which may be mutated to cause antibiotic resistance. Additionally, host-directed therapies against TB have been shown to be associated with reduced lung pathology and improved disease outcome, most likely via the modulation of host immune responses. This review will provide an update of host-directed therapies and their mechanism(s) of action against *Mycobacterium tuberculosis*.

## 1. Introduction

Tuberculosis (TB), caused by the bacillus *Mycobacterium tuberculosis* (Mtb), is one of the top 10 diseases with a deadly outcome and is ranked as the leading cause of death due to a single infectious agent, even after the emergence of the Human immunodeficiency virus/acquired immune deficiency syndrome (HIV/AIDS) epidemic [1]. The estimated number of deaths from TB is 1.3 million among HIV-negative patients and 300,000 among HIV-positive patients in 2017. Approximately 1.7 billion people are infected with Mtb, among them, 10 million people have developed symptomatic, active TB disease in 2017 [1]. The remainder of infected individuals constitute a reservoir for the development of active TB in the future. However, the vast majority of people will not develop active TB in their lifetime [2,3]. The most likely reason is that their immune system can control the TB progression, which highlights the role of host factors [4,5,6,7,8]. The morbidity and mortality caused by TB is further enhanced by immune-compromising conditions, such as coinfection with HIV, development of drug resistant Mtb strains, and the coexistence of other chronic diseases, such as diabetes, malaria, and severe viral infections [9]. 

The currently recommended curative therapy for drug-susceptible TB comprises of a 6–9 months regimen of four first line drugs: isoniazid, rifampicin, ethambutol, and pyrazinamide. According to the new guidelines from World Health Organization (WHO), more frequent patient monitoring is needed during the first two months of medication [1]. However, patient compliance decreases over time due to adverse side effects and the long duration of treatments, leading to infrequent intake and discontinuation of the regimen. This has contributed to the emergence of multi-drug resistant TB (MDR-TB) as well as totally drug resistant TB, which is a major challenge to global public health. There are approximately 500,000 new MDR-TB cases per year and the treatment success rates for MDR-TB is currently 55% compared to 85% for drug-susceptible TB [1]. MDR-TB treatment is associated with costly therapy by second-line drugs for longer periods, sometimes up to two years, which is accompanied with more complex side-effects [10]. In addition, extensively drug-resistant-TB (XDR-TB) is the most severe form that has a global treatment success rate of 30%, leading to high mortality and morbidity, especially in TB/HIV co-infected patients [11]. 

The tuberculin skin test and interferon-γ (IFN-γ) release assay are mainly used for identification of TB disease, including latent TB. If it is possible to identify the risk group of disease progression, depending on biomarkers, then treatment with HDT compounds in addition with antibiotics may be successful [12]. However, there are some TB progressors who do not show positive results for IFN-γ release assay or the tuberculin skin test. They are known as “resisters” and they maintain class-switched affinity-matured, high titers of Mtb-specific antibodies with a unique Fc profile compared with matched controls [13]. The increasing numbers of MDR-TB and XDR-TB underline the necessity to develop effective alternative or adjunctive therapeutic approaches that could speed up and improve TB treatment by targeting the host.

## 2. Immune Responses against Mtb

### 2.1. Innate Immune Responses

The innate immune responses play an important role in the protection against Mtb as it provides the first line of defense. Mtb interacts with a number of innate immune cells via surface exposed receptors, including toll-like receptors (TLRs), complement receptor (CR) 3, mannose receptor, scavenger receptors, and dendritic cell (DC)-specific intercellular-adhesion-molecule-3-grabbing nonintegrin (DC-SIGN). Engagement of these receptors leads to the induction of inflammatory responses that either can clear the Mtb infection or initiate granuloma formation [14]. The alveolar macrophages that first engulf the Mtb, provide the bacterium with its niche but are also able to neutralize the pathogen. Mtb has evolved strategies to manipulate the macrophages, allowing intracellular survival and replication. The DCs that phagocyte Mtb can also provide a replication niche and simultaneously present antigen to T-cells in the draining lymph nodes [14,15]. In contrast, Mtb has developed mechanisms to prevent the migration and antigen presentation of DCs [16]. 

A study from Madan-Lala et al., shows that Mtb impairs DC cytokine secretion, maturation, and antigen presentation through the cell envelope-associated serine hydrolase Hip1 [17]. Mtb infection is accompanied by massive influx of neutrophils at the site of infection [15]. Neutrophils can be activated in response to Mtb infection. The activated neutrophils can then kill the bacteria by using a range of antimicrobial polypeptides present in their granules, including the cathelicidin LL-37, defensins, lactoferrin, and lysozyme [15,18,19]. Neutrophils also exhibit efficient killing of Mtb through the assembly of the nicotinamide adenine dinucleotide phosphate (NADPH) oxidase in the phagosomal membrane, which leads to the generation of reactive oxygen species (ROS) in the phagosome [18]. Additionally, neutrophils have been shown to kill Mtb in a Ca^2+^-dependent manner [20]. 

Furthermore, neutrophils are able to activate macrophages through the release of azurophil granule proteins [21], neutrophil extra cellular traps (NETs) [22] and heat shock proteins [23]. Natural Killer (NK) cells have cytotoxic functions exerted through perforin, granzyme, and granulysin [15] and provide stimulatory signals to macrophages through IFN-γ during Mtb infection [24]. Moreover, NK cells also have the capacity to indirectly stimulate immune responses through the activation of macrophages with multiple signaling pathways, including generation of both reactive oxygen species (ROS) and reactive nitrogen species (RNS) [25]. Mtb-infected macrophages can be destroyed directly by NK cells, whereby the NK cells mount a pro-inflammatory response in an apoptosis-dependent manner [26]. 

A wide array of activated receptors such as NKp30, NKp44, and NKp46 [27,28,29] and the inhibitory receptors killer cell Ig-like receptors (KIRs), leukocyte Ig-like receptor (LIR), and CD94/NKG2 receptors) [30,31] as well as the cytokines interleukin (IL)-12, IL-18, and IFN-α are involved in the activation of these complex interactions between macrophages and NK cells [32]. Chowdhury et al. have reported that latent TB is associated with a higher frequency of NK cells, whereas in active TB, the corresponding number of NK cells decrease and return to base line upon clinical improvement [33]. However, the precise role of NK cells in Mtb infection is not completely clear. This underscores the complexity of the interactions between Mtb and the innate immune system.

### 2.2. Adaptive Immune Responses

After ingestion of Mtb, infected dendritic cells (DCs) and macrophages present Mtb-antigens to CD4+ T lymphocytes and initiate the activation and proliferation of lymphocytes. Infected cells also secrete different cytokines, including IL-12, IL-23, IL-7, IL-15, and tumour necrosis factor (TNF)-α, leading to attraction of more leukocytes to the site of infection [14]. Moreover, CD1-restricted T cells are activated through the presentation of glycolipid and lipid antigens by DCs [34]. It has been reviewed by Joosten et al. that donor-unrestricted T cells, comprising CD1-restricted T cells can recognize numerous antigens expressed by Mtb, including antigens expressed in BCG (*Mycobacterium bovis* bacille Calmette-Guérin). This vital role of these cells makes them potential TB vaccine targets [35,36]. 

Furthermore, γδ T cells are activated by Mtb phospho antigens and contribute to protective immunity against Mtb by producing TNF-α and IFN-γ or exerting cytotoxic activity [37]. Memory T cells are also formed upon Mtb infection. Depending on the cytokine environment, a Th1 response leads to the release of the pro-inflammatory cytokines IL-12, IL-18, and IFN-γ, enhancing the killing of intracellular Mtb through nitric oxide (NO) and ROS production in macrophages [38,39]. On the other hand, a Th2 response leads to release of IL-4, IL-5, IL-10, and IL-13, promoting B lymphocyte activation with antibody production, and anti-inflammatory macrophage responses. Th17 cells, stimulated by IL-6, IL-21, IL-23, and low levels of transforming growth factor (TGF)-β, are involved in the recruitment of innate immune cells and Th1 cells at the site of infection, secreting IL-17. 

Regulatory T cells (Treg), stimulated by IL-2 and high levels of TGF-β levels produce anti-inflammatory cytokines such as IL-10 and suppress microbicidal mechanisms in macrophages, and the activity of these cells is higher in active TB patients [40,41]. In addition, CD8^+^ cytotoxic T cells secrete perforin to lyse Mtb-infected macrophages and release granulysin in order to kill intracellular Mtb directly [42,43,44]. Since Mtb is an intracellular pathogen, the protective role for humoral immunity against tuberculosis infection remains unclear. However, studies from Lu et al. comprehensively describe antibody functions using an “unbiased antibody profiling approach”. The authors have demonstrated that individuals with latent TB infection and active TB disease exhibit diverse Mtb-specific antibody responses, such as distinct Mtb-specific IgG Fc profiles, selective binding to FcγRIII and glycosylation patterns [13,45]. 

However, antibodies might be very important in neutralization and prevention of invasion of pathogens, especially at the mucosal surfaces. TB is primarily a respiratory mucosal disease, therefore, research into the role of Mtb-directed antibodies has gained renewed interest. Notably, it has already been shown that antibodies can provide protection against TB [46,47]. Mice with depleted B-cells exhibit exacerbated immunopathology and a high neutrophil accumulation, corresponding to increased bacterial burden [48,49].

### 2.3. Granuloma Formation

The accumulation of various immune cells surrounding the infected phagocytes, in response to secreted cytokines and chemokines, results in the formation of granulomas, a hallmark of Mtb infection. The interaction between phagocyte pattern recognition receptors and Mtb antigens triggers the production of numerous pro-inflammatory cytokines, including tumor necrosis factor-α (TNF-α) and interleukin-12 (IL-12) as well as chemokines. These factors recruit and activate additional innate and adaptive immune cells from the circulation to the site of infection [50,51]. 

Although granulomas have been thought to act as a physiological barrier in preventing dissemination of infection and providing a microenvironment that facilitates the interaction between the immune cells and the pathogen. However, the granuloma can also serve as a niche, where Mtb can thrive and persist and represents the intersection of innate and adaptive immunity [52]. A mature granuloma is highly stratified, becomes vascularized, and develops a fibrotic capsule [53]. The granuloma is thought to play a major role in maintaining latent TB infection and avoiding reactivation of infection through complex immune interactions [54,55]. The hypoxic core of the granuloma may induce a dormant bacillary state, where minor or no replication of Mtb takes place, and a cellular immune response is thought to control bacterial growth during latent TB infection [56].

## 3. Endogenous Mechanisms Involved in Mtb Killing

**Figure 1:**
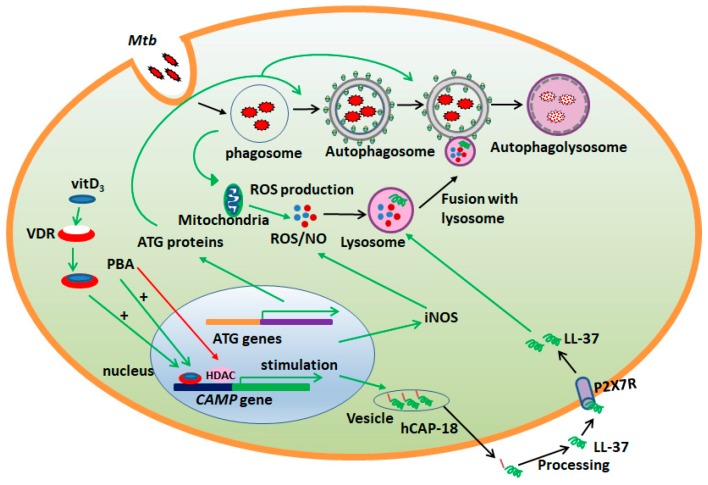
Host directed therapy (HDT) and macrophage immune defense against *Mycobacterium tuberculosis* (Mtb). Macrophages are the natural host for Mtb. Mtb prevents the formation of autophagolysosome by blocking the fusion of autophagosome with lysosome, preventing the acidification of the autophagolysosome, enabling the intracellular survival of Mtb. HDT compounds (vitamin D_3_ and phenylbutyrate) can activate the host defense pathway of autophagy, which leads to autophagolysosome formation and control of Mtb growth. Reactive oxygen species (ROS) production is increased upon Mtb infection. Vitamin D_3_ (vitD_3_) and phenylbutyrate (PBA) treatment induces the production of the antimicrobial peptide LL-37 via the recruitment of VDR (vitamin D receptor) or histone deacetylase inhibition, respectively, on the *cathelicidin antimicrobial peptide* (*CAMP*) gene (encoding hCAP-18/LL-37) promoter. Upregulation of LL-37, inducible nitric oxide synthase (iNOS) and ATG proteins in macrophages activate the autophagy process and contribute to the killing of Mtb. ATG, autophagy related; HDAC, histone deacetylase; NO, nitric oxide; P2X7R, purinergic receptor P2X7. Green arrows indicate stimulation of the process and the red arrow indicates inhibition of the process.

**Figure 2:**
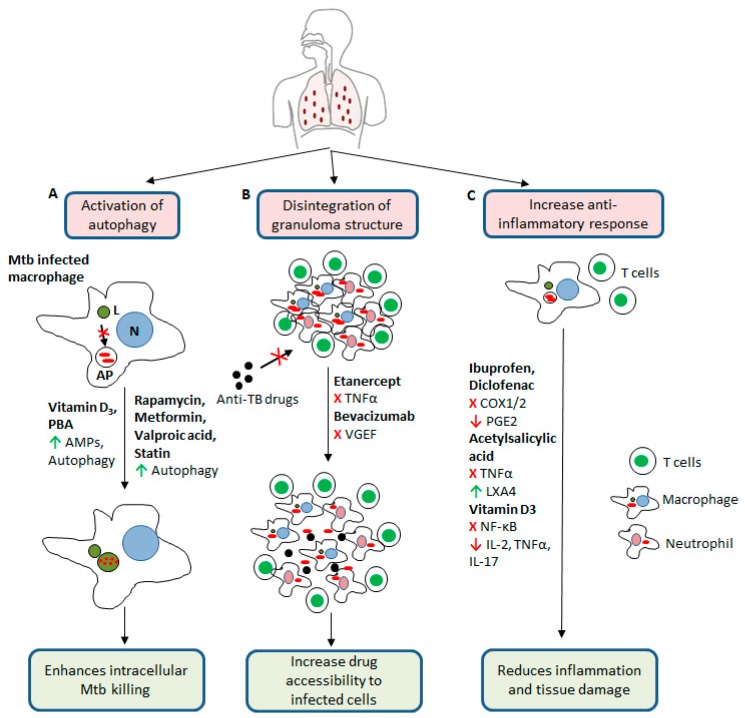
Host-directed therapy (HDT) against *Mycobacterium tuberculosis*. (A) HDT compounds upregulate the production of antimicrobial peptides and activate autophagy in Mtb infected macrophages. (B) Some HDT drugs disintegrate granuloma structure and enhance drug accessibility to the infected cells. (C) HDT agents can increase anti-inflammatory responses and suppress proinflammatory responses, which reduces inflammation and tissue damage. PBA, phenylbutyrate; AMPs, anti-microbial peptides; VEGF, vascular endothelial growth factor; COX1/2, cyclooxygenase 1/2; PGE2, Prostaglandin E2; LXA4, Lipoxin A4; NF-κB, nuclear factor-kappa B; TNFα, tumor necrosis factor alpha: AP, autophagosome; L, lysosome, N, nucleus. Green arrows indicate stimulation of the process, red arrows indicate downregulation of the process, and red crosses indicate blocking of the process.

The host immune-reaction against TB infection starts by uptake of Mtb by phagocytes, such as alveolar macrophages and dendritic cells, in the lower respiratory tract. The fate of intracellular Mtb within the phagocytes is determined by various cellular processes, including autophagy, and activation of host defense pathways, producing reactive oxygen/nitrogen species (ROS/RNS) and antimicrobial peptides (AMPs) (Figure 1 and Figure 2) [57,58].

### 3.1. Autophagy and TB

Autophagy is an evolutionary conserved, house-keeping cellular process [59]. In this process, long-lived and aggregated proteins, as well as excess and damaged organelles are targeted by a double membrane vesicle autophagosome for degradation [60]. This process is present in all cells and plays a homeostatic role, allowing the use of basic components (amino acids, and glucose) as building blocks or energy sources [61]. Autophagy plays also a key role against intracellular bacterial infection such as Mtb [57,62]. Autophagy is also critical for the regulation of a wide range of immune responses, including defenses against Mtb [63,64]. Autophagy is triggered by numerous stress signals including starvation, hypoxia, damage of intracellular organelles, and microbial infections. 

The induction of the autophagy pathway is complex; three main components are involved in the initiation of this process, the phosphoinositide 3-kinase complex 3 (PI3KC3), Unc-51-like Kinase 1 complex (ULK1), and the autophagy-related protein (ATG) complex [65]. The autophagy process begins with the formation of an autophagosome, which is a double-membrane-bound vesicle that contains cytoplasmic material or phagocytic bacteria. Various autophagy-related genes (ATGs) are involved in each step of the autophagy pathway. The ATG1/ULK complex and class III PI3K complex play essential roles in the formation of autophagosomes during the initiation stage [66]. 

These autophagosomes are non-degradative until they come in contact with the lysosomes, forming an autophagolysosome, facilitating the degradation of their contents [67]. Xenophagy is a specific type of autophagy that explains the process of delivering intracellular pathogens to the lysosomes via autophagic mechanisms [68]. It has been reported that the cellular cargo protein ubiquitin recognizes surface proteins of Mtb and activates the host xenophagy process to control intracellular growth of Mtb [69,70]. Primarily autophagy is regulated by the mammalian target of rapamycin (mTOR) complex 1 and the adenosine monophosphate-activated protein kinase (AMPK) [71,72].

Activated macrophages are capable to kill Mtb, involving autophagy (Figure 1). However, it is known that virulent strains of Mtb can prevent the fusion of the autophagosome with the lysosome and subsequent acidification of autophagolysosomal compartments by secreting antacid, 1-tuberculosinyladenosine (1-TbAd) [73]. This is a critical step for the killing of Mtb by the autophagy process, thus, evading the host immune response to survive within the macrophages [74,75,76]. The Mtb Eis (enhanced intracellular survival) protein inhibits the activation of autophagy in macrophages and cell death via a reactive oxygen species (ROS)-dependent pathway [77]. Moreover, the 6 kDa early secretory antigen target (ESAT6), a major ESAT-6 secretion system-1 (ESX-1)-mediated secretory protein, plays a crucial role in the suppression of late-stage autophagy in human dendritic cells [78,79,80]. 

As a strategy to escape host immunity, intracellular Mtb selectively modulates autophagy to survive within the macrophages, preventing infected macrophages from undergoing apoptosis [81,82]. Cytosolic Mtb DNA is recognized via the cyclic GMP-AMP synthase (cGAS) and the stimulator of interferon genes (STING)-dependent pathway, which leads to the direct ubiquitination of the bacteria, targeting bacteria to autophagy [58,83]. These two pathways have been shown to activate the Type I IFN and promote inflammation, which plays a critical role in dictating the clinical outcome of Mtb infection. The STING-dependent cytosolic pathway and autophagic receptors sequestosome 1 (SQSTM1)/p62 and nuclear dot protein 52 kDa (NDP52) play a fundamental role in the xenophagic elimination of Mtb [83]. The autophagy process is inhibited by the activation of mTOR complex [65], which is a focus for possible Mtb therapies. 

Since autophagy has emerged as a crucial protective process to restrict Mtb growth in host immune cells [84,85], it is relevant to develop host-directed therapy against tuberculosis, focusing on the activation of autophagy. We have shown that 4-phenylbutyrate (PBA) and/or vitamin D_3_ (vitD_3_), overcomes the Mtb-induced inhibition of autophagy in human macrophages via the host defense peptide LL-37 (Figure 1) [86,87].

### 3.2. Oxidative Stress and TB

Oxidative stress and antioxidative defense mechanisms play essential roles during TB infection and treatment (Figure 2) [88]. Oxidative stress can damage nucleic acids and induce protein oxidation and lipid peroxidation [89]. Mtb infection triggers macrophages to produce a respiratory burst and generate reactive oxygen species (ROS) and nitrogen intermediates [90]. Polymorphonuclear neutrophils (PMNs) are key components in the first line of defense against Mtb, and they eliminate pathogens by generating reactive oxygen species (ROS) [91,92]. Several first and second-line anti-TB drugs are administered in an inactive form and are subsequently transformed into their active form by components of the oxidative stress responses in both the host and pathogens [88]. 

For example, isoniazide (isonicotinylhydrazide) is administrated as a prodrug and is oxidatively activated by a catalase/peroxidase enzyme (KatG) present in Mtb to produce an isonicotinic acyl radical [93]. Mtb generates and secretes antioxidant enzymes to persist in the abnormal redox environment; these enzymes include superoxide dismutase (SOD) and KatG. Most of the Mtb strains have the ability to produce enhanced intracellular survival (Eis) proteins, which can detect ROS and respond in a counteractive way [94]. 

In addition, Mtb has also developed various mechanisms to survive during high oxidative stress of the host, including increased bacterial NADH/NAD^+^ ratio, peroxiredoxin, superoxide dismutases, and catalases. Increased mycobacterial NADH/NAD^+^ ratio, through mutation of *ndh*, which encodes a type II NADH dehydrogenase, exhibits co-resistance to isoniazid and ethionamide [95]. Mtb strains that show resistance to isoniazid also exhibit more resistance to ROS compared to the wild-type Mtb (CDC1551 and CB3.3) strains [96,97]. It has also been observed that these strains have increased resistance to peroxide and acidified nitrite molecules [96,98]. Moreover, Mtb retains proteasome (peroxiredoxin) that can recognize, repair, and remove oxidatively altered or damaged proteins [99]. Mtb also generate altered peroxidase systems to maintain its virulence, including the expression of alkyl hydroperoxide reductase subunit C (AhpC) [100]. Along with ROS, nitric oxide (NO) contributes to host defense against Mtb infection in humans. It has been reported that, alveolar macrophages from healthy controls infected with Mtb produce NO, and this production of NO correlates with the intracellular growth inhibition of Mtb [101]. Additionally, some studies have revealed that alveolar macrophages are able to kill mycobacteria and that these antimycobacterial activities are dependent on iNOS expression [102,103]. Moreover, increased iNOS expression and pulmonary NO production have been reported in alveolar macrophages and PBMCs from TB patients as compared to healthy controls [104,105,106]. These findings suggest a significant role of NO in host defense against mycobacterial infection.

## 4. Host-Directed Therapy (HDT)

Tuberculosis is often associated with immunodeviation of the host. Ineffective or sub-optimal treatments lead to increase Mtb-associated morbidity, in particular, infections with MDR and XDR Mtb strains, before starting efficacious treatment according to drug-resistance profiles. Since very few new potent anti-TB drugs are in the pipeline in the near future, host directed therapy (HDT) could be an attractive additive approach to restore or to enhance host immunity. The aim would be to maximize bacterial killing, while minimizing inflammatory tissue damage together with conventional anti-TB drugs [107]. 

HDT includes agents that are not microbicidal per se, instead, these agents modulate host immunity, combating Mtb and may additively or synergistically enhance the activity of the anti-TB drugs. These new approaches are referred to as adjunctive therapy [108]. Different clinical studies have demonstrated that host-directed adjunctive therapies acting via novel mechanisms of action have the potential to decrease the duration of treatment, reduce transmissibility, and improve outcomes in MDR-TB [109]. Most of the HDTs target highly conserved host signaling pathways, which eliminates the risk of emergence of resistance.

### 4.1. Antimicrobial Peptides and TB

Antimicrobial peptides (AMPs) are positively charged peptides, comprising 12–50 amino acids. By virtue of positively charged amino acids, such as arginine and lysine, the AMPs have a net positive charge. AMPs are amphipathic in structure, with hydrophobic and hydrophilic regions that facilitate their interaction with bacterial membranes [110]. In mammals there are two main families of endogenous AMPs, cathelicidins and defensins. They can reduce the infectious load by different mechanism of actions, and modulate host immune responses [111]. The single human cathelicidin, LL-37, is expressed in immune cells, such as neutrophils, monocytes, macrophages, mast cells, and epithelial cells. The expression of LL-37 is constitutive or inducible depending on the host condition and the specific cell types [112]. AMPs, such as LL-37, kill mycobacteria by disrupting membrane functions and by interfering with cell wall formation [113].

In a TB infection, Mtb downregulates the expression of LL-37 in human macrophages, and this reduction can be counteracted by treating the infected macrophages with vitD_3_, phenylbutyrate (PBA) or a combination (Table 1) [86]. The active form of vitamin D_3_, 1,25(OH)_2_D_3_, is an immunomodulatory compound and sodium phenylbutyrate (PBA) is a histone deacetylase inhibitor, already on the market for another indication [114]. To use these two compounds with standard anti-TB drugs could be an attractive approach to treat TB [115,116]. 

Vitamin D and PBA are potent inducers of LL-37 in a variety of cell types [86,117,118]. Accordingly, the combination of vitamin D and PBA are in restricting intracellular growth of drug susceptible Mtb in macrophages by the expression of LL-37 and LL-37 dependent autophagy (Figure 1) [86,119,120]. However, vitamin D may influence genes related to cellular metabolism in addition to the induction of T cell tolerance and anti-microbial defense [121]. Notably, PBA is a histone deacetylase inhibitor, which opens up the chromatin, leading to access to other genes that are not related to anti-microbial defense [122].

### 4.2. Vitamin D and TB

The non-skeletal function of vitamin D has gained intense interest over recent years due to its ability to modulate immune responses [137]. Several studies have reported the positive association between active TB and low levels of serum vitamin D at diagnosis and during the course of treatment with anti-TB drugs [138,139]. Vitamin D inhibits the proliferation of Mtb inside the macrophages through stimulation of the innate immune responses during the infection [140]. In addition, vitamin D has also an impact on the differentiation of naïve T cells to regulatory T cells, and regulates the function of cytotoxic T cells, indicating a potential role in adaptive immunity during infections [141]. 

Liu et al. reported that TLR dependent upregulation of the vitamin D receptor and the vitamin D-1-hydroxylase gene in macrophages is associated with induction of LL-37 and subsequent killing of intracellular Mtb [142]. In the same study it was reported that individuals with increased susceptibility to tuberculosis have low 25-hydroxyvitamin D_3_, and these low levels are not enough to support production of LL-37 mRNA. In line with this result, low levels of LL-37 were observed in situ in granulomas of patients with chronic TB and severe vitamin D deficiency [143]. 

Another study has reported that treatment of THP-1 cells with anti-TB drugs is associated with upregulation of LL-37 only in the presence of active vitamin D, supporting the assumption of beneficial effects of vitamin D supplementation during standard anti-TB therapy. In addition, vitamin D has also been shown to enhance the anti-inflammatory cytokine response and suppress the pro-inflammatory response, which limits the excessive tissue damage during active TB (Figure 2) [144].

### 4.3. Other HDT Related Compounds and TB

Rapamycin inhibits mTOR kinase activity, which in turn activates autophagy. Macrophages use autophagy to restrict Mtb replication (Table 1, Figure 1 and Figure 2) and present antigens to other immune cells [57]. However, the use of rapamycin as HDT is limited in TB because of obstacles related to adverse effects. Another drawback of using rapamycin in TB-infection is that rapamycin is metabolized by the hepatic enzymes CYP3A4, which is induced by the key first-line drug rifampicin [123,124]. Another candidate of HDT is metformin (Table 1), which is a common drug for treating diabetes mellitus type 2. In an in vitro study metformin treatment was found to be associated with reduced Mtb survival, increased production of mitochondrial reactive oxygen species and induced phagosome-lysosome fusion in infected macrophages [125]. In mice, metformin treatment is associated with enhanced specific immune responses and reduced chronic inflammation and lung pathology [125]. Additionally, metformin treatment is associated with improved control of Mtb and decreased disease severity in diabetic patients with TB [125].

Two anticonvulsant drugs, carbamazepine and valproic acid (Table 1) have shown promising effects by clearing Mtb in infected primary human macrophages through inducing mTOR-independent autophagy [126]. The same study reported that carbamazepine treatment is associated with enhanced clearance of *Mycobacterium marinum* in zebrafish and reduced bacterial burden, improved lung pathology and stimulation of adaptive immunity in a mice model [126]. The authors suggested that enhancement of autophagy by repurposed drugs could be a potential therapy for the treatment of MDR-TB [126]. Another drug-class that has been proposed for HDT are the statins (Table 1), cholesterol-lowering drugs, normally used for reducing the risk of coronary disorders and hypercholesterolemia. Interestingly, peripheral blood mono-nuclear cells (PBMC) and monocyte derived macrophages (MDMs) from patients with familial hypercholesterolemia receiving statin therapy are less susceptible to Mtb and exhibit reduced bacterial burden compared to healthy donors [127]. It was demonstrated that statin treatment helped to lower cholesterol levels within phagosomal membranes, which in turn activated host-induced autophagy and lead to protection against TB [127].

Attenuating the inflammatory responses through HDT might be another interesting strategy, as aggressive inflammatory immune responses in active TB lead to host tissue damage, necrosis and cavitation [145]. Non-steroidal anti-inflammatory drugs (NSAIDs), including diclofenac and ibuprofen (Table 1) could alleviate inflammatory responses and lung pathology, as indicated by different preclinical studies in mice [128,129]. Another proposed HDT candidate is acetylsalicylic acid (Table 1), which can induce lipoxin A4, a key regulator that maintains TB progression. It has been shown that acetylsalicylic acid improve treatment outcome by suppressing neutrophil migration and TNF-α production [130,131]. In addition, TNF-α plays a key role in granuloma formation and maintenance of the integrity of the granuloma. Etanercept, a TNF-α inhibitor (Table 1) has been associated with improved treatment outcome and reduced lung pathology [132,133,134]. However, there is an increasing risk of TB reactivation, when using TNF-α inhibitors [132,133]. Similar to anti-TNF-α, bevacizumab treatment of infected rabbits has been shown to promote vascular normalization, to improve small molecule delivery, and to decrease hypoxia in TB granulomas [135]. This morphological change of granulomas can improve tissue penetration and efficacy of current anti-TB regimens (Figure 2) [135,136].

### 4.4. Additional Micronutrients and TB

Vitamin A deficiency increases the risk of being infected with TB. It has been shown that up to 10 fold increases of TB cases among household contacts of patients with tuberculosis in Lima, Peru are deficient in vitamin A [146]. In a study of Moroccan populations a significantly lower plasma retinol concentration in TB patients compared to healthy controls was observed [147]. In a multinational case-cohort study, it has been reported that both Vitamin A and D deficiencies are associated with the incident of TB cases in HIV-Infected patients, initiating antiretroviral therapy [148]. Zinc deficiency has also been observed in patients with tuberculosis compared to controls [149,150]. Similarly, low levels of vitamins A, D, and E have been observed in patients with TB than in controls [151]. Therefore, many micronutrients might be used as potential HDTs, and the management of TB needs to consider multiple micronutrient deficiencies in addition to protein-energy malnutrition.

### 4.5. Clinical Trials and Host Directed Therapy

A randomized, double-blind and placebo-controlled trial in Bangladesh by Mily et al. demonstrated that daily supplementation of phenylbutyrate (PBA), vitamin D_3_ (vitD_3_), or PBA+vitD_3_ for 2 months to patients with drug-susceptible pulmonary TB enhances the efficacy of standard chemotherapy [120]. In particular it was shown that the odds of sputum culture being negative at week 4 were 2–4 times higher in the PBA+vitD_3_ or vitD_3_ supplemented groups compared to a placebo. The proportion of sputum culture negative at week 4 was 1.5–2 times higher in the supplementation groups compared to the placebo group. A similar study in Ethiopia demonstrated that daily adjunct supplementation of PBA+vitD_3_ for 4 months ameliorated clinical TB symptoms and disease specific-complications. However, the authors of these studies did not observe any beneficial effect on bacterial clearance in sputum [119]. 

In the Ethiopian study it was speculated that the adjunctive supplementation could be beneficial for certain high-risk groups of the population, for example those patients with vitD_3_ deficiency, or with immunodeficiency diseases, or for those suffering from MDR-TB or latent TB. VitD_3_ as adjunct therapy in TB treatment has been utilized in several clinical trials, and the primary clinical outcomes are not always in line with the expected protective effects. A recent systematic review by Wallis et al. has discussed eight clinical studies where vitD_3_ was used as adjunctive therapy in TB treatment [152]. Among these eight studies only two of them showed significant microbiologically positive effects of vitD_3_. One study demonstrated positive effects, when supplemented with large vitD_3_ doses in TB patients with seasonal vitamin D insufficiency, and that treatment time was reduced from 6 to 4 months. 

The authors acknowledge that it is difficult to compare the studies because they are different in relation to patients’ genetic backgrounds, baseline vitD_3_, administered dose, dosing schedule, study endpoints, and the quality of the reported results [152]. Another recent randomized and placebo-controlled trial with a high dose of vitD_3_ (four biweekly doses of 3.5 mg) during TB treatment has been conducted in Mongolia [153]. The authors did not observe any positive effects of vitD_3_ as adjunct therapy on the primary outcome that was time to sputum culture conversion. Interestingly, the authors found that sputum smear conversion was more rapid in the treatment group and also that favorable immunomodulatory functions were detected in the treatment group. Additionally, it was found that the intervention effect was modified by single-nucleotide polymorphisms in the vitamin D receptor (VDR) and 25-hydroxyvitamin D 1a-hydroxylase (CYP27B1). The null effect on the primary outcome might be related to the use of an intermittent bolus dose instead of a daily recommended dose. 

As Martineau et al. have shown in a systematic review, daily and weekly dosing of vitD_3_ supplementation was superior to bolus-dosing with regard to enhancement of the host response against respiratory pathogens [154,155]. A recent review article with a meta-analysis of data from individual participants in eight randomized controlled trials has shown that vitD_3_ supplementation accelerated sputum smear conversion [156]. The influence of vitD_3_ supplementation on sputum culture conversion was not observed in drug-sensitive pulmonary TB, instead the positive effect was observed in patients with MDR-TB [156].

The positive effects of the vitD_3_ and PBA supplementation in the clinical trial in the Bangladeshi population may be explained by the higher expression of LL-37 in immune cells [120]. In a follow-up study, it was shown that vitD_3_ and PBA exhibit immunomodulatory functions [87]. They observed reduced levels of pro-inflammatory cytokines in the supernatants of peripheral blood mononuclear cells (PBMC), lower expression of endoplasmic reticulum stress-related genes and higher expression of the autophagy marker LC3 in macrophages from TB patients, supplemented with vitD_3_, PBA, or a combination, compared to the placebo group [87].

## 5. Conclusions

The development of novel treatment options for drug-susceptible and drug-resistant TB is urgently required for the eradication of the TB epidemic in many parts of the world. In particular, for the treatment of MDR-TB and XDR-TB, HDT is emerging as an attractive strategy, harnessing immune responses against Mtb instead of direct bactericidal effects. HDT has the potential to modulate host factors that may overcome disease-associated morbidities, also in combination with less effective second-line anti-TB drugs. Combined use of HDT with standard anti-TB drugs may potentially lead to shortening the timeframe of anti-TB therapy. Moreover, HDT has similar effects against drug-susceptible and drug-resistant strains of Mtb, and there is a lower risk of developing resistance against HDT compounds. Although significant advances have been made during the last few years, there is a pressing need to develop relevant in vitro and in vivo models to assess immunomodulatory functions of HDT-compounds alone or in combination with standard anti-TB drugs. In addition, more clinical trials are needed to evaluate the efficacy and safety of potential HDT compounds. At the same time, it is of the highest priority to find reliable biomarkers to monitor treatment efficacy.

## Figures and Tables

**Table 1:** HDT related compounds and their host target pathways related to Mtb-control.

Compounds	Target Pathways and Mechanisms	Reference
Active vitamin D	Binds to vitamin D receptor and induce antimicrobial peptide (AMP)-expression, LL-37 dependent autophagy induction, immunomodulation	Bekele et al. [119] Martineau et al. [115] Mily et al. [120] Rekha et al. [86,87]
Phenylbutyrate	Histone deacetylase inhibitor, induction of AMPs	Coussens et al. [116] Mily et al. [120] Rekha et al. [86,87]
Rapamycin	Inhibition of mammalian target of rapamycin (mTOR), activation of autophagy	Palucci et al. [123] Torfs et al. [124]
Metformin	Reduce phosphorylation of mTOR and P^70s6k^, activation of autophagy	Singhal et al. [125]
Carbamazepine, Valproic acid	Activation of autophagy	Schiebler et al. [126]
Statin	Inhibition of cholesterol in phagosomal membrane, activation of autophagy	Parihar et al. [127]
Nonsteroidal anti-inflammatory drugs (NSAIDs) (diclofenac and ibuprofen)	Inhibition of cyclooxygenase 1 and 2, reduce prostaglandin E2 (PGE2) production	Dutta et al. [128] Vilaplana et al. [129]
Acetylsalicylic acid	Induce lipoxin A4 (LXA4) production, suppress neutrophil migration and tumour necrosis factor (TNF)-α production	Rizvi et al. [130] Byrne et al. [131]
Etanercept	TNF-α neutralization, disruption of granuloma, reduce lung pathology	Skerry et al. [132] Bourigault et al. [133] Wallis et al. [134]
Bevacizumab	Vascular endothelial growth factor (VEGF) neutralization, vascular normalization, reduce hypoxic fractions	Datta et al. [135] Oehlers et al. [136]
